# Clonal spread of *Mycobacterium monacense* strains among patients with extrapulmonary mycobacteriosis

**DOI:** 10.1007/s10096-025-05318-y

**Published:** 2025-10-28

**Authors:** Christoffel Opperman, Aysha Ahmed, Marianna De Kock, Claudia Spies, Michael Biggel, Tristen Lourens, Sarishna Singh, Yonas Ghebrekristos, Robin Warren, Wynand Goosen, Giovanni Ghielmetti

**Affiliations:** 1 National Health Laboratory Service, Green Point TB-Laboratory, Cape Town, South Africa; 2 SAMRC Centre for Tuberculosis Research, Division of Molecular Biology and Human Genetics, Faculty of Health Sciences, Stellenbosch University, Cape Town, South Africa; 3 Division of Medical Microbiology, Department of Pathology, Faculty of Health Sciences, University of Cape Town, Cape Town, South Africa; 4 Institute for Food Safety and Hygiene, Vetsuisse Faculty, University of Zurich, Zurich, Switzerland; 5 Department of Microbiology and Biochemistry, Faculty of Natural and Agricultural Sciences, University of the Free State, Bloemfontein, South Africa; 6 Section of Veterinary Bacteriology, Institute for Food Safety and Hygiene, Vetsuisse Faculty, University of Zurich, Zurich, Switzerland; 7 Block D Old City Hospital Complex Green Point TB-Laboratory, 1 Portswood Road, Green Point, Cape Town, 8005 South Africa

**Keywords:** Mycobacterium monacense, Nontuberculous mycobacteria, Whole-genome sequencing, Antimicrobial susceptibility testing, Extrapulmonary, Plasmid

## Abstract

**Background:**

This study presents the phylogenetic and antimicrobial susceptibility characterization of *Mycobacterium monacense*, a rare nontuberculous mycobacterium (NTM), cultured from clinical extrapulmonary samples.

**Methods:**

Eight *Mycobacterium monacense* isolates were identified between 2019 and 2023 in the Western Cape province of South Africa. Whole-genome sequencing (WGS) was applied to assess phylogenetic relatedness, identify virulence factors, and characterize the resistome of the isolates. Antimicrobial susceptibility testing (AST) was performed using the GenoType NTM-DR line probe assay (LPA), Sensititre minimum inhibitory concentrations (MIC) plates, and the proportional method based on critical concentrations. Spatial distribution of cases was mapped using ArcGIS software.

**Results:**

Spatiotemporal distribution patterns indicated the presence of circulating clones confined within specific geographical areas. Plasmids coding for ferredoxin and cytochrome P450 genes were identified in one cluster, which notably lacked the chromosomal *mbtH *gene involved in siderophore biosynthesis for iron acquisition. In contrast, isolates grouped in a second cluster harbored the *mbtH* chromosomal gene but lacked these plasmid-associated elements. LPA and broth microdilution showed that all *Mycobacterium monacense* isolates were susceptible to aminoglycosides, fluoroquinolones, and macrolides, but generally exhibited elevated MICs against β-lactam antibiotics. Phenotypic AST indicated that drugs commonly used to treat *Mycobacterium tuberculosis* complex (MTBC), namely bedaquiline, linezolid, and rifampicin, are effective against *Mycobacterium monacense*.

**Conclusion:**

*Mycobacterium monacense* in extrapulmonary cultures accentuates the need for improved diagnostics and enhanced clinical awareness of infections with rare NTM. WGS highlights the potential significance provided by plasmid-encoded genes. Current treatment regimens for MTBC exhibit therapeutic efficacy against *Mycobacterium monacense* isolates.

**Supplementary Information:**

The online version contains supplementary material available at 10.1007/s10096-025-05318-y.

## Introduction

*Mycobacterium monacense* is a rapidly growing, yellow-pigmented, scotochromogenic nontuberculous mycobacterium (NTM) that was first isolated in 1998 in Germany from a bronchial lavage specimen [1]. Since its initial identification, several cases of extrapulmonary site infections have been reported, including bacteremia [2], osteomyelitis [3], and soft tissue infections [4, 5]. The bacteria have been isolated globally, with reports from Africa [6], Asia [2, 7–9], Europe [10], North America [4], and South America [11], highlighting its widespread presence.

The European Reference Laboratory Network for Tuberculosis (ERLTB-Net) has recently advocated for the development of novel diagnostic and therapeutic approaches to enhance understanding of NTM and facilitate the translation of research findings into improved clinical care [12]. With advancements in sequencing techniques for the identification of NTM, *M. monacense* has emerged as an increasingly recognized infection [6]. Despite the rise in its detection, very little is known about its genetic acquisition of single nucleotide polymorphisms (SNPs), its geographical distribution, or its role as a pathogen in human disease. In this context, the present study describes a unique cohort of clinical *M. monacense* isolates recovered from extrapulmonary cultures between 2019 and 2023. Whole-genome sequencing (WGS) and antimicrobial susceptibility testing (AST) were performed to characterize their genomic features and antimicrobial resistance profiles.

## Methods

### Ethics approval

An ethical waiver of informed consent was provided by the Human Research Ethics Committee of Stellenbosch University (SU HREC reference number: S22/10/191). Additionally, the University of Cape Town’s Human Research Ethics Committee authorized the collection and storage of rare NTM cultures (UCT HREC reference number: R013/2023). Institutional approval was granted by the National Health Laboratory Service (NHLS) to genetically and phenotypically investigate the extrapulmonary cultures (PR2232714).

### Isolation and confirmation of *Mycobacteriummonacense*

*Mycobacterium monacense* was isolated from extrapulmonary cultures previously screened using multilocus targeted deep amplicon-based sequencing with Oxford Nanopore Technology (ONT) [6]. The current numbering follows the biorepository system for rare NTM isolates stored at the Green Point Complex, TB Laboratory, in Cape Town, South Africa, and is consistent with previously published work [6]. To confirm the presence of *M. monacense*, 100 µl (µL) of Mycobacteria Growth Indicator Tube (MGIT) culture, suspected to harbor the strain, were inoculated onto an NTM Elite agar plate (bioMérieux, France) and incubated aerobically at 30 °C, following the manufacturer’s instructions [13]. Yellow-pigmented, scotochromogenic colonies were selected, and pure colonies were obtained through subsequent sub-culturing. Acid-fast bacilli were confirmed via Ziehl-Neelsen staining of all positive cultures. From 14 potential MGIT cultures identified as genetically containing *M. monacense* [6], eight viable strains were successfully sub-cultured on the NTM Elite agar for further investigation.

Additional verification of *M. monacense* cultures was performed through amplification and Sanger sequencing of the *hsp65* gene, as previously described [6]. The *M. monacense* isolates from the cultures were confirmed using both *rpoB* and *hsp65* gene sequencing in a previous study [6]. In the current study, a single gene target (*hsp65*) was selected for confirmation of subculture. Purified amplicons were submitted to the Central Analytical Facility at Stellenbosch University (Cape Town, South Africa) for sequencing. The resulting consensus sequences were analyzed using the National Center for Biotechnology Information (NCBI) nucleotide Basic Local Alignment Search Tool (BLASTn). Identification was considered reliable when both the similarity index and percentage coverage exceeded 99%, aligning with the reference *M. monacense* DSM 44,395 (CP035734.1) type strain sequence available in GenBank [14].

### Whole-genome sequencing using Oxford nanopore technologies sequencing platform

DNA was extracted using a modified version of the DNeasy Blood and Tissue Kit (Qiagen, Hilden, Germany) [6] and subsequently used for WGS. DNA concentrations were determined using the Qubit Double-Stranded DNA High Sensitivity Assay Kit (Life Technologies, California, USA). WGS was performed according to the manufacturer’s instructions [15]. Briefly, 400 nanogram of native genomic DNA were used as the starting volume. DNA underwent end-repair using the NEBNext Ultra II End Repair/dA-Tailing Module (New England Biolabs, Massachusetts, USA). DNA extracts were barcoded with the ONT Native Barcoding Kit v14 (Oxford, UK) and pooled in equimolar concentrations to create a unified library. Native adapters were ligated using the NEB Blunt/TA Ligase Master Mix and Quick Ligation Module (New England Biolabs), and the final library (35 femtomoles) were loaded onto a single PromethION flow cell with >4600 active pores and sequenced on a PromethION 2 Solo device (ONT). Cluster groups were confirmed by re-sequencing newly extracted DNA from one randomly selected isolate from each group separately, using individual R10.4.1 flow cells, each containing >1250 active pores (ONT). Re-sequencing was performed on the MinION Mk1C device (ONT).

### Bioinformatics and construction of phylogenetic trees

Eight sequencing datasets were generated. Super-accurate base calling [v4.3.0, 400 base pairs (bps)], de-multiplexing, and barcode trimming were performed using Dorado (v7.4.13). Quality control of the raw reads was assessed using FastQC (v0.11.9) [16] and pycoQC (v2.5.0.23) [17]. To evaluate potential sample contamination, taxonomic classification of sequencing reads was performed using Kraken2 (v2.1.3) [18] with the standard database (7/14/2025). The classification results were subsequently visualized with KronaTools (v2.5) [19]. Short reads (< 1000 bp) or reads with a Phred quality score below 12 were excluded using Nanoq (v0.10.0). Reads were subsampled with rasusa (v2.0.0) [20] to a target coverage of 100×. ONT-only assemblies were generated from the subsampled reads with flye (v2.9.2) (nanohq option, one iteration) [21] and circular contigs rotated using Dnaapler (v0.7.0) [22]. Assemblies were then polished with Medaka (v2.1.0; https://github.com/nanoporetech/medaka) in conjunction with the r1041_e82_400bps_bacterial_methylation model.

To provide phylogenetic context, determination of closest type strain genomes was done by comparing the newly generated assemblies against all type strain genomes available in the Type (Strain) Genome Server (TYGS) database [23] using the MASH algorithm [24] and ten type strains with the smallest MASH distances were chosen for downstream comparison (https://tygs.dsmz.de). Briefly, for the phylogenomic inference, all pairwise comparisons among the set of genomes were conducted using the Genome BLAST Distance Phylogeny approach (GBDP). Hundred distance replicates were calculated each. Digital DDH values and confidence intervals were calculated using the recommended settings of the GGDC 4.0 [25, 26]. The resulting intergenomic distances were used to infer a balanced minimum evolution tree with branch support via FASTME 2.1.6.1 including SPR post processing [27]. Branch support was inferred from 100 pseudo-bootstrap replicates each. The trees were rooted at the midpoint and visualized with PhyD3 [28]. A custom whole genome multilocus sequence typing (wgMLST) scheme was created using pyMLST [29] and the annotated complete genome of *M. monacense* strain JCM 15,658 (RefSeq GCF_010731575.1_ASM1073157v1). In order to provide genetic context to the newly sequenced strains, available genomes of *M. monacense* from the NCBI were included in the analysis. A core genome MLST (cgMLST; 4,131 loci) scheme comprising genes present in at least 95% of the strains was generated. Finally, a matrix of cgMLST distances was computed from the cgMLST database and defined as the number of different alleles between each pair of two strains, omitting the missing data. The obtained matrix was visualized using the pheatmap package (https://github.com/raivokolde/pheatmap).

Reads-based variant calling was performed by aligning ONT reads to the *Mycobacterium monacense* JCM 15,658 reference genome (ASM1073157v1; GCF_010731575.1) using Minimap2 v2.28-r1209 [30], with the map-ont option for long-read data. Variant calling was conducted with bcftools v1.15.1, using bcftools mpileup to compute genotype likelihoods and bcftools call to identify SNPs and generate VCF files [31]. Variants were filtered using vcffilter to retain positions with a minimum quality score of 20. To create a SNP matrix across all samples, the resulting VCF files were processed with a custom Python script adapted from the vSNP pipeline [32]. Briefly, each VCF was parsed into a Pandas DataFrame containing key fields (CHROM, POS, REF, ALT, QUAL, AC, DP, MQ), and an absolute position identifier (CHROM: POS) was generated for each variant. A union of all unique SNP positions across samples was then compiled, and a sample-by-position matrix was constructed, where each cell indicated the corresponding allele or reference base at that genomic position. Plasmid sequences were identified using MOB-Suite and typing was performed using MOB-typer [33].

Assembled contigs were annotated using Bakta [34] (v1.8.2, DB: v5.0 - Light) and screened for antimicrobial resistance associated genes with AMRfinderPlus (v3.11.2) and ABRicate (v0.8.10; https://github.com/tseemann/abricate) using the NCBI database, ResFinder 3.0, and the Comprehensive Antibiotic Resistance Database (CARD) v3.1.0 [35–37], with a threshold for the identification of acquired genes of 65 % minimum length and 95% identity. Candidate virulence genes were identified using AMRfinderPlus v3.11.2 and the virulence factor database (vfdb) from ABRicate with relaxed thresholds of ≥ 90% sequence coverage and ≥ 80% nucleotide identity [38, 39].

### Clinical, laboratory data, and geographic mapping

Available laboratory results and clinical information were retrieved from the laboratory information system (TrackCare, Version L2016, InterSystems, Sydney, Australia). Additionally, the residential addresses of the cases were geospatially mapped using ArcGIS^®^Pro version 2.0 (Environmental Systems Research Institute, Redlands, CA) to explore possible geographic patterns in the distribution of the cases. The residential addresses were de-identified using a numbering system that was not linked to any patient identifiers.

### GenoType NTM-DR line probe assay

The GenoType^®^NTM (Non-Tuberculous Mycobacteria) – DR (Drug Resistant) (Bruker, Billerica, MA, USA) line probe assay (LPA) version 1.0 was employed to detect resistance to macrolides and aminoglycosides using a DNA-DNA reverse hybridization method. The method evaluates both wild-type and mutation bands. The assay was performed in accordance with the manufacturer’s instructions [40]. DNA was extracted from subcultures using the Genolyse^®^ kit version 1.0 (Bruker, Billerica, MA, USA). The provided PCR mastermix, which contains primers and Taq polymerase, required the addition of 5 µL of extracted DNA to achieve a final volume of 50 µL. Amplification conditions included one cycle at 95 °C for 15 min, followed by 10 cycles at 95 °C for 30 s and 65 °C for 2 min. Subsequently, 20 cycles were performed with 25 s at 95 °C, 40 s at 50 °C, and 40 s at 70 °C. Finally, one cycle was conducted at 70 °C for 8 min. Hybridization was carried out using an automated GT Blot 48 device (Hain Lifescience, Nehren, Germany). A negative control (5 µL molecular biology grade water) and positive controls were included: *Mycobacterium abscessus*, which contains the *erm* [41] *T28* gene mutation, and a *Mycobacterium spp*., which carried both the *rrl* Mut1 (A2058C) and *rrs* Mut1 (A1408G) gene mutations.

### Sensititre minimum inhibitory concentration susceptibility plates

The Thermo Scientific™ Sensititre™ MIC Susceptibility plates (Waltham, Massachusetts, USA) for rapidly growing mycobacteria were used to determine the minimum inhibitory concentration (MIC) values for the following antibiotics: amikacin, amoxicillin/clavulanic acid, cefepime, cefoxitin, ceftriaxone, ciprofloxacin, clarithromycin, doxycycline, imipenem, linezolid, minocycline, moxifloxacin, tigecycline, tobramycin, and trimethoprim/sulfamethoxazole. Sensititre™ demineralized water was inoculated with a 0.5 McFarland *M. monacense* solution using a Sensititre™ nephelometer (Waltham, Massachusetts, USA). Following this, 50 µL of the suspension was transferred to a Sensititre™ cation-adjusted Mueller-Hinton broth to achieve an inoculum of 5 × 10⁵ colony-forming units per milliliter. A volume of 100 µL was then transferred to each well of the plate. The plates were incubated in non-carbon dioxide conditions at 35 ± 2 °C. To ensure the detection of inducible macrolide resistance, the incubation period was extended to 14 days as recommended by the manufacturer. The plates were read according to the manufacturer’s instructions using a Sensititre™ Vizion with the Thermo Scientific™ Sensititre™ SWIN™ Software system (Waltham, Massachusetts, USA) and manually with a reverse mirror. *Mycobacterium smegmatis* American Type Culture Collection (ATCC) 19,420 was used as a positive control as specified by the manufacturer [41].

### Adapted phenotypic proportional drug susceptibility testing

Phenotypic drug susceptibility testing (DST) was performed on *M. monacense* isolates using the BACTEC MGIT 960 liquid culture system (Becton Dickinson, NJ, USA) at the NHLS, Green Point, TB Laboratory, Cape Town, South Africa. The test employed the 1% proportion method, utilizing critical drug concentrations consistent with World Health Organization standards for *Mycobacterium tuberculosis*: 1.0 micrograms per milliliter (µg/mL) for bedaquiline and linezolid, 0.5 µg/mL for rifampicin, and 0.1 µg/mL for isoniazid. Although these concentrations are standardized for *Mycobacterium tuberculosis*, they were extrapolated and applied to *M. monacense* in the absence of species-specific guidelines in this in-house method. The method was adapted from testing procedures used for *M. tuberculosis*, beginning with the preparation of an inoculum from solid culture media (Lowenstein–Jensen) less than 14 days old. Colonies were suspended aseptically in MGIT broth to a turbidity equivalent to a 0.5 McFarland standard. This suspension was then diluted in 4 mL of sterile saline and mixed thoroughly. For each drug-containing and growth control tube, 800 µL of BACTEC MGIT960 SIRE supplement (oleic acid, albumin, dextrose, catalase, and polyoxyethylene stearate) was added, followed by 100 µL of the respective antimicrobial agent. A growth control tube was prepared by diluting 100 µL of the original suspension in 10 mL of sterile saline (1:100 dilution), from which 500 µL was inoculated. In each drug-containing tube, 500 µL of the undiluted organism suspension was added. All tubes were then loaded into the MGIT960 instrument for automated incubation and susceptibility analysis. Drug-containing tubes were analyzed once the growth control (no antimicrobial) reached 400 growth units. A drug-containing tube that demonstrates ≥ 100 growth units at this point is typically considered to harbor an organism resistant to the drug in question.

## Results

### Isolation and confirmation of *Mycobacteriummonacense*

All isolates included in this study were confirmed as *M. monacense* based on partial *hsp65* gene sequencing, showing > 99% coverage and identity. Interestingly, partial sequencing of the *hsp65* gene revealed five and three single-nucleotide variants (SNVs) compared to the *M. monacense* type strain DSM 44,395 (GenBank accession CP035734.1) for isolates classified as cluster 1 and cluster 2, respectively. Two SNVs differentiated the two clusters: one at position 693,244 (C→T) and another at position 693,514 (T→C) in the reference genome CP035734.1.

### Whole-genome sequencing using Oxford nanopore technologies sequencing platform

**Fig. 1:**
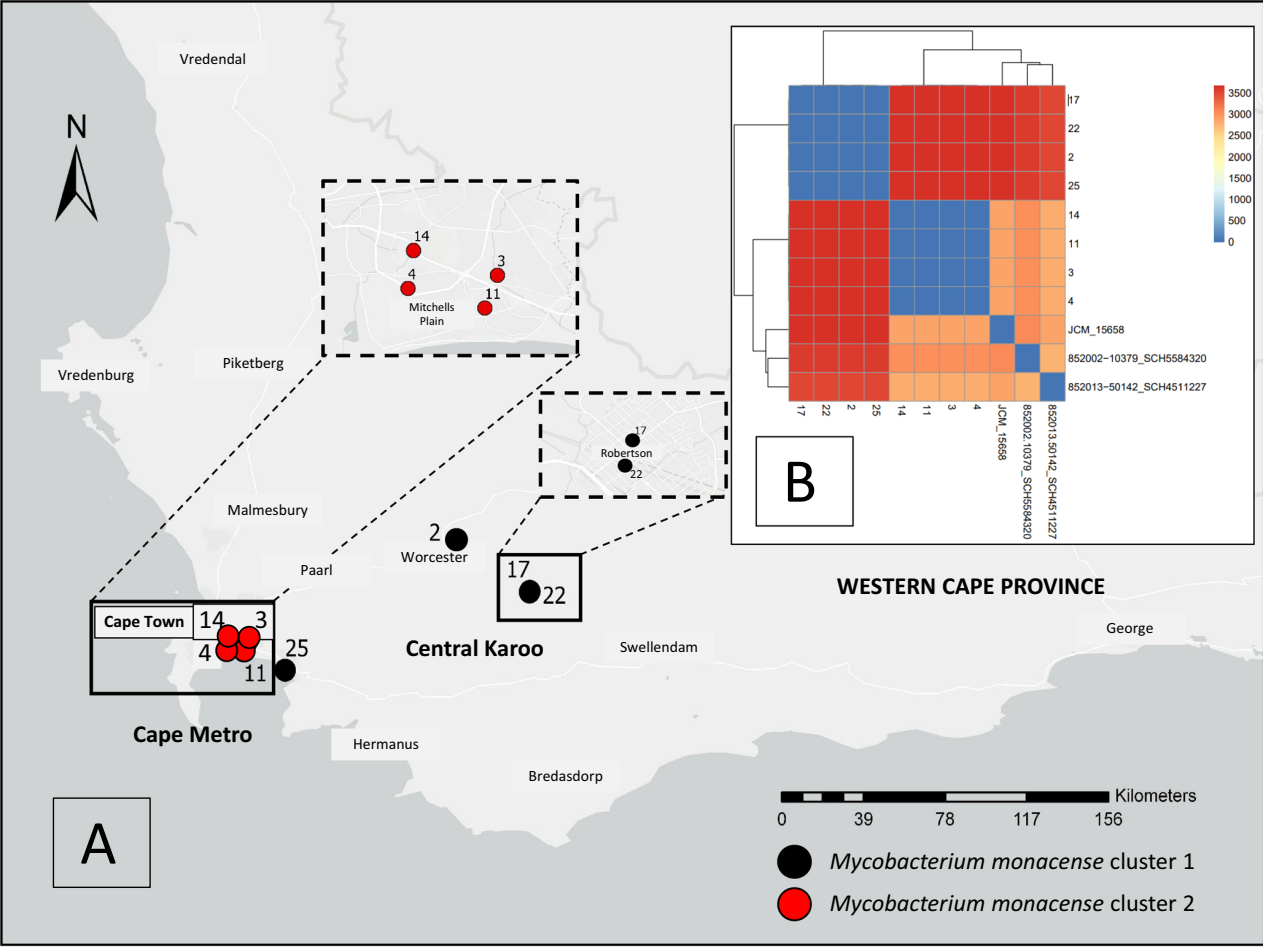
Spatial distribution of *Mycobacterium monacense* isolates in the Western Cape province, South Africa. Cluster 1 (isolates 2, 17, 22, and 25) was identified in the Central Karoo district, while cluster 2 (isolates 3, 4, 11, and 14) was located in the Cape Metro area (panel **A**). Core genome multilocus sequence typing (cgMLST) was performed, incorporating the two publicly available genomes from the National Center for Biotechnology Information (NCBI; 852002 − 10379_SCH5584320 and 852013 − 50142_SCH4511227) along with the reference strain, to provide contextual information regarding genetic distances (panel **B**)

**Fig. 2:**
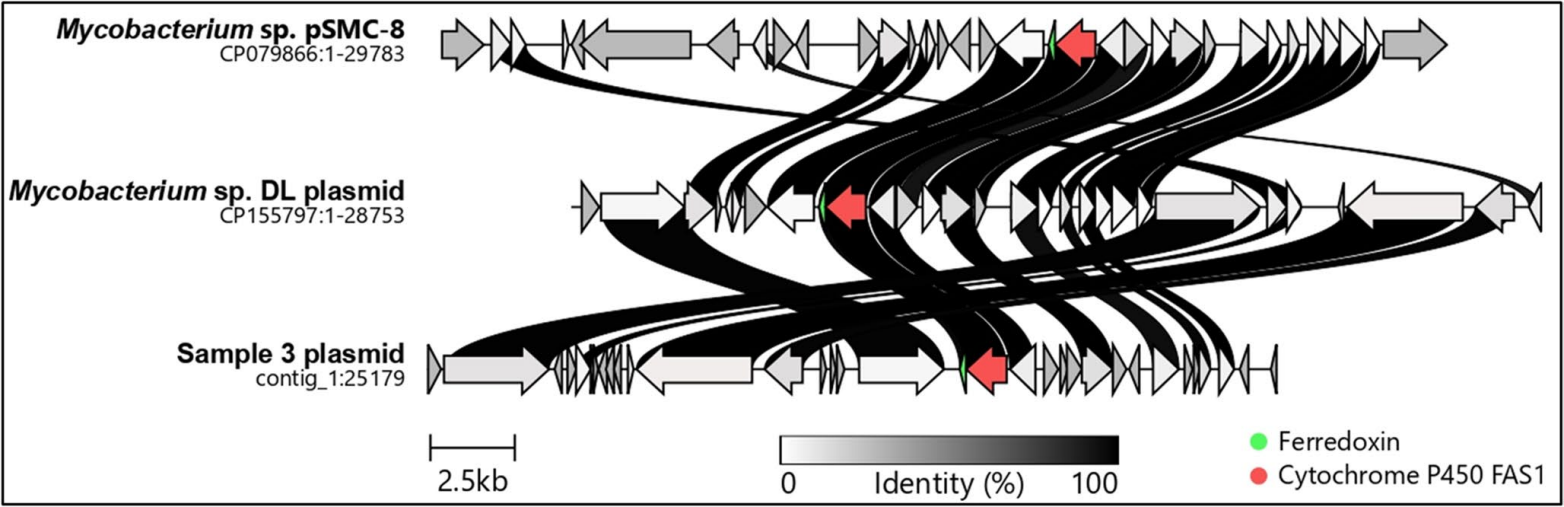
Comparative plasmid analysis of a cluster 2 isolate. Graphical representation of the plasmid identified in cluster 2 (samples 3 representing the cluster isolates 3, 4, 11, 14), compared with the most closely related plasmids, *Mycobacterium spp*. pSMC-8 and *Mycobacterium spp*. DL plasmid, as retrieved from the National Center for Biotechnology Information (NCBI) database. Annotation revealed conserved genes encoding ferredoxin (green arrow) and cytochrome P450 (red arrow), while the majority of the remaining open reading frames were annotated as hypothetical proteins. The greyscale intensity of the connections between genes reflects the percentage (%) of sequence homology between the plasmids

After filtering for reads with a quality score of > Q12 and length > 1000 bp, an average of 1.1 million reads per sample were retained, ranging from a minimum of 771,265 to a maximum of 1,700,493 reads. The median read length was 3,742 bp, and the median read quality score was 20.2 (Supplementary Table 1). Assembled genomes comprised three contigs for isolates lacking plasmids and one additional circular contig of 25,191 base pairs for isolates containing the plasmid. The average chromosomal size was 5,825,284 base pairs (Supplementary Table 2). Two genetically distinct clusters were identified: cluster 1 (isolates 2, 17, 22, 25) and cluster 2 (isolates 3, 4, 11, 14), separated by > 8000 SNPs. Multilocus sequence typing, based on 4,131 core genes, demonstrated close relatedness within each cluster (Fig. 1B). Plasmids were identified exclusively within cluster 2, carrying replication initiator protein *repA*, a ParA family partitioning protein, and a putative relaxase belonging to rep_cluster_1566. Based on MOB-suite analysis, the plasmids were classified as non-mobilizable. Comparative analysis using the NCBI database showed that these plasmids have a high similarity [identities 13,119/13,176 (99%) and 11,953/11,953 (100%)] to *Mycobacterium spp*. pSMC8 (CP079866.1) and the *Mycobacterium spp*. DL (CP155797.1) plasmid (Fig. 2). Annotation confirmed the presence of genes encoding ferredoxin and cytochrome P450, while the majority of other open reading frames were classified as hypothetical proteins (Fig. 2).

The resistome analysis showed that cluster 1 carried *blaS*, *aac(2′)-Ib*, *tet(V)*, and *arr* genes, whereas cluster 2 contained *blaS*, *aac(2′)-Ib*, *tet(V)*, and *rox* (Table 1). None of these genes were located on plasmids. These resistance determinants are associated with reduced susceptibility to β-lactams, aminoglycosides, tetracyclines, and rifamycins. Several virulence-associated genes were identified across both clusters, including *relA* (GTP pyrophosphokinase involved in bacterial persistence), *ideR* (iron-dependent regulator crucial for iron homeostasis), *icl* (isocitrate lyase supporting intracellular survival), and *phoP* (a transcriptional regulator modulating virulence and lipid biosynthesis). Additionally, *mbtH*, which encodes a siderophore-associated protein involved in iron acquisition, was uniquely present on the chromosome of cluster 1 isolates (Table 2).

**Table 1:** Phenotypic and genotypic antimicrobial susceptibility profile of Mycobacterium monacense clusters

Sensititre™ MIC susceptibility plates							
		Mode MIC (µg/mL) for Cluster 1*. Plates evaluated on Day 7 (Day14†)	Broth microdilution interpretive criteria for rapidly growing mycobacteria‡	Mode MIC (µg/mL) for Cluster 2*. Plates evaluated on Day 7 (Day14)		Broth microdilution interpretive criteria for rapidly growing mycobacteria	*Mycobacterium smegmatis *(ATCC 1940) MIC (µg/mL). Quality control *Mycobacterium*
Drug	**Drug-class**						
Amikacin	Aminoglycoside	≤ 1 (≤ 1)	Susceptible	≤ 1 (≤ 1)		Susceptible	≤ 1
Amoxicillin/Clavulanic acid	Penicillin + Beta-lactamase inhibitor	>64/32 (>64/32)	No breakpoint	>64/32 (>64/32)		No breakpoint	>64
Cefepime	Cephalosporin (fourth-generation)	>32 (≥32)	No breakpoint	>32 (≥32)		No breakpoint	>32
Cefoxitin	Cephalosporin (second-generation)	≥64 (≥128)	Resistant	≥64 (≥64)		Resistant	128
Ceftriaxone	Cephalosporin (third-generation)	>64 (≥64)	No breakpoint	>64 (≥64)		No breakpoint	>64
Ciprofloxacin	Fluoroquinolone	≤ 0.12 (≤ 0.12)	Susceptible	≤ 0.12 (≤ 0.12)		Susceptible	0.5 §
Clarithromycin	Macrolide	0.25 (0.25)	Susceptible	≤ 0.06 (≤ 0.06)		Susceptible	4
Doxycycline	Tetracycline	2 (2)	Intermediate	1 (0.25)		Susceptible	2
Imipenem	Carbapenem	>64 (≥64)	Resistant	>64 (≥64)		Resistant	>64
Linezolid	Oxazolidinone	≤ 1 (≤ 1)	Susceptible	≤ 1 (≤ 1)		Susceptible	2
Minocycline	Tetracycline	4 (4)	Intermediate	4 (4)		Intermediate	4
Moxifloxacin	Fluoroquinolone	≤ 0.25 (≤ 0.25)	Susceptible	≤ 0.25 (≤ 0.25)		Susceptible	≤ 0.25
Tygicycline	Glycylcycline	1 (1)	No breakpoint	0.5 (0.5)		No breakpoint	1
Tobramycin	Aminoglycoside	2 (2)	Susceptible	2 (2)		Susceptible	≤ 1
Trimethoprim/Sulfamethoxazole	Antifolate (sulfonamide + folate synthesis inhibitor)	≤ 0.25/4.75 (≤ 0.25/4.75)	Susceptible	≤ 0.25/4.75		Susceptible	≤ 0.25
GenoType^®^NTM-DR line probe assay							
		**Gene mutations for Cluster 1**	**Gene mutations for Cluster 2**	**Interpretation of gene markers for drug susceptibility**	***Mycobacterium*** ** spp.**		***Mycobacterium abscessus***
Gene	**Drug-class**				Positive control		Positive control
*rrl*	Macrolide	Absent	Absent	Susceptible	*rrl *Mut1 (A2058C) Present		Absent
*rrs*	Aminoglycosides	Absent	Absent	Susceptible	*rrs *Mut1 (A1408G) Present		Absent
*erm[41] C28*	Macrolide	Absent	Absent	Susceptible	Absent		Absent
*erm[41] T28*	Macrolide	Absent	Absent	Susceptible	Absent		Present
Whole-genome sequencing (Oxford Nanopore Technologies next generation long-read sequencing)							
Gene	**Gene description**	**Drug-class**	**Cluster**	**Alignment length**	**Percentage (%) coverage¶**		**Percentage (%) identification¶**
*blaS*	Class A Beta-lactamase	Beta-lactam	Cluster 1	291	98.29		67.35
*aac(2')-Ib*	Aminoglycoside N-acetyltransferase	Aminoglycoside	Cluster 1	192	97.44		75.00
*tet(V)*	Tetracycline efflux major facilitator superfamily (MFS) transporter	Tetracycline	Cluster 1	419	98.81		79.71
*Arr*	Nicotinamide adenine dinucleotide (NAD^+^) rifampin Adenosine diphosphate (ADP)-ribosyltransferase	Rifamycin	Cluster 1	137	95.80		83.94
*blaS*	Class A Beta-lactamase	Beta-lactam	Cluster 2	287	97.95		68.99
*aac(2')-Ib*	Aminoglycoside N-acetyltransferase	Aminoglycoside	Cluster 2	192	97.44		75.00
*tet(V)*	Tetracycline efflux MFS transporter	Tetracycline	Cluster 2	419	98.81		79.00
*rox*	Rifampin monooxygenase	Rifamycin	Cluster 2	472	99.16		69.28
Proportional phenotypic drug susceptibility testing#							
Drug (critical concentration)**	**Drug-class (action)**	**Cluster 1 (range of growth units) ††**	**Growth unit interpretation with most likely susceptibility**††	**Cluster 2 (range of growth units) ††**	**Growth unit interpretation with most likely susceptibility**‡‡		***Mycobacterium tuberculosis*** ** H37Rv (Growth units)**
Bedaquiline (1.0 µg/mL)	Diarylquinoline (Adenosine triphosphate synthase inhibitor)	0	Susceptible	0	Susceptible		Susceptible (0)
Linezolid (1.0 µg/mL)	Oxazolidinone (Protein synthesis inhibitor)	0-3	Susceptible	0-1	Susceptible		Susceptible (0)
Isoniazid (1.0 µg/mL)	Isonicotinic acid hydrazide (Inhibits mycolic acid synthesis)	34-2256	Resistant	63-1077	Resistant		Susceptible (0)
Rifampicin (0.5 µg/mL)	Rifamycin (Ribonucleic acid polymerase inhibitor)	0	Susceptible	0	Susceptible		Susceptible (0)

ATCC: American type culture collection; MIC: minimum inhibitory concentration; µg/mL: micrograms per milliliter

* Cluster 1 cultures: 2,17,22, 25; Cluster 2 cultures: 3,4,11,14

† Prolonged incubation of Sensititre susceptibility MIC plates may reveal inducible antibiotic resistance phenotypes

‡ Susceptibility Testing of Mycobacteria, Nocardiae, and Other Aerobic Actinomycetes. 2nd ed. Wayne (PA): Clinical and Laboratory Standards Institute (CLSI); 2011

§ The quality control MIC range for *Mycobacterium smegmatis* ATCC 19420 on Sensititre™MIC susceptibility plates for ciprofloxacin is 0.25–1.0 µg/mL

¶ Antimicrobial resistance-associated genes were identified using AMRfinderPlus and ABRicate, applying sequence coverage thresholds >95% and identity >65%. Genes detected included those present in the National Center for Biotechnology Information (NCBI) database, ResFinder 3.0, and the Comprehensive Antibiotic Resistance Database (CARD v3.1.0)

# Resistance is inferred when the proportion of colonies growing on the drug-containing medium exceeds 1% relative to growth on a drug-free control, indicating that a significant subpopulation of the isolate is capable of proliferating despite the presence of the antimicrobial agent

** The proportional method was originally developed and standardized for *Mycobacterium tuberculosis* complex and should be interpreted as a guideline when applied to *Mycobacterium monacense*

†† Growth units are interpreted when the control well reaches a value of 400

‡‡ Isolates exhibiting growth unit values less than or equal to 100 in the presence of the antimicrobial agent are considered susceptible

**Table 2:** Clinical and culture characteristics of the Mycobacterium monacense patient cohort

Culture	Cluster group	Clinical sample type	Sample collection year	Age in years	Sex	Working diagnosis during admission	One year all-cause mortality outcome	Additional laboratory results	Human immunodeficiency virus	Sample collection site hospital/clinic	Virulence factors*
2	1	Fine needle aspiration	2021	40	Female	Malignancy	Alive	Histological examination revealed no evidence of malignancy.	Positive	Hospital	*relA*, *ideR*, *icl*, *phoP*, *mbtH*
3	2	Cerebrospinal fluid	2021	15	Female	Meningitis	Alive	Cerebrospinal fluid was acellular, with glucose and protein concentrations within normal reference ranges.	Negative	Hospital	*relA*, *ideR*, *icl*, *phoP*
4	2	Synovial knee fluid	2021	50	Male	Synovitis or Bursitis	Alive	No bacterial growth or crystals detected in synovial (knee) fluid culture.	Negative	Clinic	*relA*, *ideR*, *icl*, *phoP*
11	2	Synovial knee fluid	2020	28	Male	Septic arthritis or ankylosing spondylitis	Alive	*Staphylococcus aureus* cultured from synovial (knee) fluid.	Negative	Hospital	*relA*, *ideR*, *icl*, *phoP*
14	2	Left lung empyema	2019	54	Female	Bacterial empyema	Alive	Exudative fluid obtained from left lung empyema; no bacterial organisms cultured.	Positive	Hospital	*relA*, *ideR*, *icl*, *phoP*
17	1	Urine	2022	41	Female	Uncomplicated urinary tract infection	Alive	*Escherichia coli* isolated from urine, microscopy showed abundant leukocytes.	Positive	Clinic	*relA*, *ideR*, *icl*, *phoP*, *mbtH*
22	1	Peritoneal fluid aspirate	2022	47	Female	Malignancy or *Mycobacterium tuberculosis* complex	Alive	Histopathological analysis identified a steroid cell tumor.	Positive	Hospital	*relA*, *ideR*, *icl*, *phoP*, *mbtH*
25	1	Right lung pleural fluid	2023	50	Male	Pneumonia complicated with a pleural effusion	Alive	Exudative pleural fluid from the right lung; cultures yielded no bacterial growth.	Not tested	Hospital	*relA*, *ideR*, *icl*, *phoP*, *mbtH*

*relA* (GTP pyrophosphokinase involved in bacterial persistence), *ideR* (iron-dependent regulator crucial for iron homeostasis), *icl* (isocitrate lyase supporting intracellular survival), *phoP* (a transcriptional regulator modulating virulence and lipid biosynthesis), *mbtH* (siderophore-associated protein involved in iron acquisition)

* Virulence genes were retained if they demonstrated ≥ 90% sequence coverage and ≥ 80% nucleotide identity compared to the virulence factor database (vfdb version 2025–04–05)

### Construction of phylogenetic trees

**Fig. 3:**
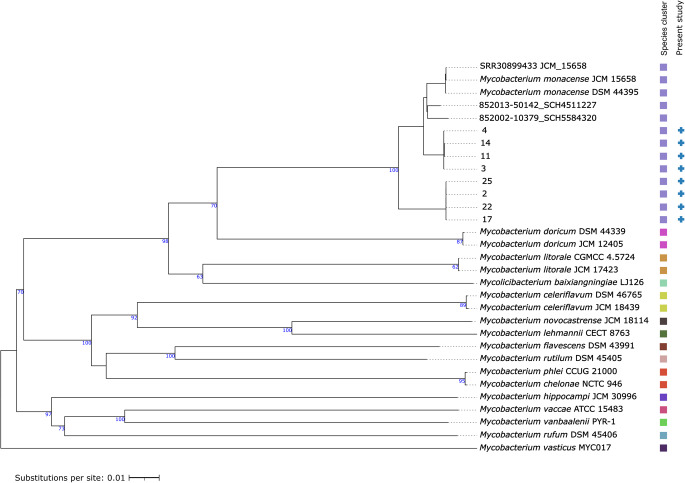
Phylogenetic tree inferred with FastME using the Genome BLAST Distance Phylogeny approach (GBDP) distances calculated from genome sequences. The branch lengths are scaled in terms of GBDP distance formula d5. The numbers above branches are GBDP pseudo-bootstrap support values > 60% from 100 replications. Two distinct *Mycobacterium monacense* clusters were identified: cluster 1 (isolates 2, 17, 22, 25) and cluster 2 (isolates 3, 4, 11, 14). Isolates SCH5584320 and SCH4511227 represent two previously reported cases from South Africa. Reference strains *Mycobacterium monacense* JCM 15658 (Japan Collection of Microorganisms) and *Mycobacterium monacense* DSM 44395 (Deutsche Sammlung von Mikroorganismen und Zellkulturen – German Collection of Microorganisms and Cell Cultures) were included from international genomic databases

Phylogenetic analysis revealed two distinct *M. monacense* clusters (Fig. 3). Cluster 1 comprised isolates 2, 17, 22, and 25, while Cluster 2 included isolates 3, 4, 11, and 14. Isolates within each cluster were clonal, and major branches were supported by bootstrap values exceeding 60%, with branch lengths indicating clear genetic separation between clusters.

### Clinical, laboratory data, and geographic mapping

Clinical isolates included in this study (Table 2) were obtained from a diverse range of specimen types collected between 2019 and 2023. The types of clinical cultures processed comprised fine needle aspiration (2021), cerebrospinal fluid (2021), synovial knee fluid (2020 and 2021), left lung empyema (2019), urine (2022), peritoneal fluid aspirate (2022), and right lung pleural fluid (2023). Of the eight *M. monacense* isolates cultured (Table 2), five were from female patients (62.5%), and four of the seven patients tested (57.1%) were human immunodeficiency virus (HIV)–immunocompromised. The average patient age was 40.6 years (range: 15–54 years). All patients were alive at one-year follow-up. Spatial mapping based on WGS identified two distinct clusters: one in the Central Karoo district (cluster 1) and another in the Cape Metro area (cluster 2), both within the Western Cape province of South Africa (Fig. 3A). One isolate (5) from cluster 1 was mapped to the Cape Town area. However, patient movement could not be traced, and it remains unclear whether this individual had travelled to the Central Karoo region at the time of sample collection or infection. The Central Karoo lies approximately 200 km northeast of Cape Town.

### GenoType NTM-DR line probe assay

All eight *M. monacense* strains exhibited conjugate band formation, indicating a positive substrate reaction and efficient conjugate binding. The presence of a universal control band confirmed the existence of a high guanine-cytosine (G + C) content organism. Additionally, the species-specific band 4 was observed in all strains, along with the control loci for the *rrl* (targeting *23 S rRNA*) and *rrs* (targeting *16 S rRNA*) genes, which are associated with resistance to macrolides and aminoglycosides, respectively. No mutations were detected in the *rrl*, *rrs*, or *erm* gene regions (Table 1).

### Sensititre MIC susceptibility plates

Broth microdilution assays revealed susceptibility to amikacin (mode MIC: ≤1), ciprofloxacin (mode MIC: ≤0.12), clarithromycin [(cluster 1-mode MIC: 0.25); (cluster 2-mode MIC: ≤ 0.06)], linezolid (mode MIC: ≤1), moxifloxacin (mode MIC: ≤0.25), tobramycin (mode MIC: 2), and trimethoprim/sulfamethoxazole (mode MIC: ≤ 0.25/4.75) in both genetic clusters, according to Clinical and Laboratory Standards Institute (CLSI) criteria. While no established breakpoints exist for beta-lactams in this NTM, the MICs for these antibiotics were consistently elevated (mode MIC: >32). Additionally, no inducible resistance was observed following prolonged incubation for up to 14 days in the macrolides for both clusters (Table 1).

### Adapted phenotypic proportional drug susceptibility testing

Both clusters demonstrated the absence or low growth units in the presence of bedaquiline [clusters 1 and 2 [(susceptible, 0 growth units)], linezolid [cluster 1 (susceptible, 0 to 3 growth units); cluster 2 (susceptible, 0 to 1 growth units)], and rifampicin [clusters 1 and 2 (susceptible, 0 growth units)]. In contrast, elevated growth rates were observed in the presence of isoniazid [cluster 1 (34 to 2,256 growth units); cluster 2 (63 to 1,077 growth units)], suggestive of reduced susceptibility or resistance to this agent (Table 1).

## Discussion

We present new insights into the genetic relatedness, pathogenic potential, and antimicrobial susceptibility of *M. monacense* in extrapulmonary infections. The cases were associated with two genetically and geographically distinct clonal clusters, based on clinical isolates collected between 2019 and 2023. The two clusters were separated by > 8,000 SNPs. The absence of genetic variation within each cluster suggests either a slow evolutionary rate or recent acquisition from a common environmental or clinical source.

It is not uncommon for *Mycobacteria* to have slow mutation rates. In a study by Kaewprasert et al.., (2022) [42], clonal strains of *Mycobacterium abscessus subspecies abscessus* (MAB) and *massiliense* (MMAS), responsible for persistent infections, exhibited low genomic variation over time, with SNP differences ranging from 0 to 7 in MAB and 0–14 in MMAS, when isolated between 1 and 659 days apart. The authors observed a time-dependent decline in mutation rates for both subspecies. For isolates collected over 180 days apart, MMAS showed a mutation rate of 2.89 ± 1.02 SNPs per 5 megabase (Mb) genome/year, compared to MAB [(0.82 ± 0.83; (*p* = 0.01)] [42]. In addition, the MTBC is well-documented in the literature to exhibit a mutation rate of approximately 0.2 to 0.5 SNPs/genome/year [43]. Given that the clonal isolates were collected over several years, assuming that *M. monacense* also exhibits a slow SNP acquisition rate is reasonable, despite being a fast-growing NTM. Alternatively, the observed clustering may reflect a shared source of infection or a common environmental exposure. However, due to limitations in tracing patient movement within the community, we were unable to identify a specific point source for NTM acquisition.

NTM are ubiquitously found in the environment. While human-to-human transmission is relatively uncommon, zoonotic and environmental transmission has been well documented [44]. The reservoirs and modes of acquisition for NTM, especially in cases of community transmission, remain poorly understood and are frequently unresolved during investigations [12]. Environmental sources, particularly water, are often implicated in clonal outbreaks. For instance, *Mycobacterium porcinum* was isolated from 24 patients over a five-year period, with molecular analysis revealing that 92% of clinical isolates were clonal and matched strains recovered from contaminated hospital and household water, including cases presumed to be community-acquired [45]. Moreover, NTM are known to form biofilms in plumbing systems and water reservoirs, supporting their potential role as point sources for proximal dissemination across wide geographic areas [46].

Plasmids are generally absent in MTBC and *Mycobacterium leprae*, although they are frequently present in several clinically important species of NTM [47]. Genomic studies have demonstrated that NTM genomes often contain multiple plasmids, with an average of two to five plasmids identified in plasmid-bearing strains [47]. Despite their prevalence, the NTM plasmidome remains poorly characterized, and many plasmid-encoded genes are still annotated as hypothetical proteins, which reflects the limited understanding of their functional roles. A recent NTM study highlighted that, among 17,547 protein-coding genes analyzed from 196 plasmids, 8,168 (46.5%) were annotated as hypothetical proteins [47]. The plasmids we identified had a high similarity to *Mycobacterium spp*. pSMC8 and the *Mycobacterium spp.* DL plasmid. Strain SMC-8 was isolated from a human sputum sample in Seoul, South Korea, on 11 July 2020, from a patient with lung disease (CP079866.1). In contrast, strain DL was collected in 2017 from the spleen of *Dicentrarchus labrax* (European sea bass) in western Greece, following isolation via cell culture (CP155797.1). Diricks et al., 2025 also demonstrated that plasmid SMC-8has been identified in multiple NTM species [47]. In the present study, strains within cluster 1 were found to lack plasmids but carried the *mtbH* gene on the chromosome. This gene encodes mycobactin, a siderophore involved in the uptake of extracellular iron into the cytoplasm [48]. Mycobactin plays a critical role in iron acquisition, particularly under conditions of limited iron availability such as those encountered within macrophages [49]. In contrast, strains in cluster 2 did not possess the *mtbH* gene but instead carried plasmids that encode ferredoxins and cytochrome P450 enzymes. Ferredoxins are iron–sulfur proteins that participate in electron transfer processes and are involved in a wide range of biological redox reactions [50]. In the context of cytochrome P450, ferredoxins act as electron donors and support the catalytic activity of these enzymes. The differences observed between the two clusters may indicate the presence of distinct adaptive mechanisms. In the absence of functional data, we hypothesize that the plasmidome and chromosomal genes may contribute to the following adaptive advantages. Strains that rely on siderophore-mediated iron acquisition may be better adapted to intracellular environments where iron is scarce [51]. In contrast, strains that carry plasmid-encoded redox systems involving ferredoxins and cytochrome P450 may be more suited to environments rich in lipids and oxidative stress, such as granulomatous tissue [52, 53]. In such settings, these systems support the metabolism of complex lipids including mycolic acids and contribute to the detoxification of reactive oxygen species, thereby enhancing bacterial survival and persistence.

The observed discordance between antimicrobial resistance profiles obtained through WGS, LPAs, and broth microdilution susceptibility testing was evident, indicating the shortcomings of current computational tools in reliably predicting resistance. This was highlighted by Niemann and colleagues, who, in their analysis of NTM plasmids, found that amino acid identities to resistance proteins based on AMRFinder and a reference database were often low, typically below 55% [47]. These findings emphasize the need to integrate WGS data with complementary in vitro approaches, such as phenotypic drug susceptibility testing, and in vivo models, including antibiotic-treated, infected mice, to accurately validate the functional relevance of resistance markers in NTM [47]. All isolates exhibited susceptibility to aminoglycosides, macrolides, and fluoroquinolones, on the NTM-DR LPAs and Sensititre plates, supporting the inclusion of these agents as key components of definitive treatment regimens, consistent with case-based reports in the literature [54].

It is important to recognize that in vitro antimicrobial susceptibility testing does not account for activity against intracellular organisms or bacteria embedded within biofilms [55]. These assays are designed to assess antimicrobial efficacy against planktonic cells under controlled laboratory conditions, which may not accurately reflect the complex microenvironments encountered in clinical infections. Additionally, no variation in colony morphotypes was observed among the solid-media cultured isolates, such as the well-documented smooth and rough phenotypes of *M. abscessus* [55], which can significantly influence antimicrobial susceptibility within a single strain population. Some antimicrobials, such as beta-lactams, do not have established interpretive breakpoints for *Mycobacterium monacense* as defined by the CLSI. As a result, while elevated MICs were observed, these findings should be interpreted with caution, as they are suggestive, but not confirmatory of resistance.

Phenotypic DST revealed that the isolates were susceptible to bedaquiline, linezolid, and rifampicin, based on critical concentrations extrapolated from World Health Organization guidelines for MTBC [56]. Bedaquiline is currently a key component of all-oral regimens for drug-resistant tuberculosis. Beyond its established role in TB management, bedaquiline has shown promising activity as a repurposed agent against multiple NTM species, particularly those from extrapulmonary sites [57]. The current findings suggest that *M. monacense* may be at least partially responsive to treatment regimens used for MTBC, indicating potential therapeutic relevance in cases of co-infection involving both MTBC and *M. monacense*. The importance of treating MTBC-NTM coinfections is highlighted by a recent study demonstrating significantly worse outcomes for MTBC-NTM co-infected patients compared to both NTM-only *p* and tuberculosis-only *p* groups [58]. Additionally, the study also revealed an increased mortality risk in the co-infected group *p* [58]. Identifying such co-infections within pragmatic laboratory workflows warrants further investigation in future prospective studies [59]. It is important to appreciate that no clinical trials currently exist for the treatment of MTBC-NTM co-infections or for *M. monacense* specifically, therefore, the optimal treatment regimen remains unknown.

We acknowledge several limitations of this study. First, although potential virulence factors were identified, transcriptomic analysis was not performed, and phenotypic antimicrobial susceptibility testing was limited to the drugs available in our public diagnostic laboratory. The GenoType NTM-DR assay is not formally validated for *M. monacense*; therefore, resistance results should be interpreted with caution when applying the assay to this species [40]. We were unable to present any data, including treatment information, that was not available in the laboratory information system. Since only the GenoType Mycobacterium Common Mycobacteria (CM) and Additional Species (AS) LPAs (Bruker, Billerica, Massachusetts, United States of America) are available in South African public diagnostic laboratories, which are unable to identify *M. monacense*, the results were reported to the clinicians at the time as *Mycobacterium spp*. As a result, the infection may not have been treated effectively, especially from the extrapulmonary sites. However, no deaths were reported within the 1-year all-cause mortality follow-up period. No environmental sampling from water sources was performed to confirm the presence of *M. monacense*, this endeavour will form part of future investigations. Lastly, screening and genomic characterization of unidentified *Mycobacterium spp*. from clinical cultures collected in the Western Cape province, South Africa, including for *M. monacense*, remain ongoing in our laboratory.

## Conclusion

The identification of *M. monacense* from extrapulmonary body sites accentuates the need for increased clinical awareness and enhanced diagnostic capabilities for NTM, particularly for less frequently isolated species. Curating comprehensive databases of virulence factors and resistomes in NTM, including *M. monacense*, is essential to ensure alignment between WGS and phenotypic AST methods. The plasmidome of *M. monacense* likely contributes to its environmental adaptability. Current treatment regimens for MTBC provide at least partial activity against *M. monacense* and could be effective in cases of co-infection.

## Supplementary Information

Below is the link to the electronic supplementary material.

Supplementary Material 1

Supplementary Material 2

## Data Availability

The data supporting the findings of this study are accessible through a controlled access repository and can be obtained from the corresponding author (CJ Opperman) upon reasonable request. Institutional approvals from the National Health Laboratory Service, Stellenbosch University, and the University of Cape Town is required for access. In addition, the sequencing datasets presented in this study were submitted to the European Nucleotide Archive (ENA) under project reference number PRJEB98678, https://www.ebi.ac.uk/ena/browser/view/PRJEB98678.
